# Current prevalence and therapeutic strategies for porcine *Streptococcus suis* in China

**DOI:** 10.1128/aem.02160-24

**Published:** 2025-02-25

**Authors:** Ruoyi Lv, Wenjing Zhang, Zhigang Sun, Xiaohui Si, Hong Dong, Xiaoye Liu

**Affiliations:** 1 Beijing Key Laboratory of Traditional Chinese Veterinary Medicine, Beijing University of Agriculture 74684 , Beijing, China; 2 Animal Science and Technology College, Beijing University of Agriculture 74684 , Beijing, China; 3 Beijing Traditional Chinese Veterinary Engineering Center, Beijing University of Agriculture 74684 , Beijing, China; Universidad de los Andes, Bogotá, Colombia

**Keywords:** porcine *Streptococcus suis*, prevalence, drug resistance, therapeutic strategies, multitarget drugs

## Abstract

Porcine *Streptococcus suis* is a zoonotic bacterial pathogen that poses serious threats to both human and animal health. *S. suis* is ubiquitously transmitted from the swine industry to the environments and human communities. However, the ambiguous epidemiological patterns and the escalating risk of antimicrobial resistance render *S. suis* infections a considerable challenge. Here, we review the current prevalence of *S. suis* infection worldwide, including identified bacterial strains, routes of infection, and transformation of resistance genes. This comprehensive overview of the prevalent patterns in *S. suis* offers detailed insights into therapeutic approaches for porcine infections and alternative strategies to address emerging resistant strains, highlighting potential multitarget prevention and treatment options to combat *S. suis* infection.

## PORCINE *STREPTOCOCCUS SUIS*

Porcine *Streptococcus suis* is a gram-positive pathogenic bacterium that frequently causes sudden death, meningitis, arthritis, and endocarditis in swine (1). As a significant zoonotic pathogen, *S. suis* predominantly targets the porcine respiratory tract and can be transmitted to humans via direct contact with infected animals, consumption of raw or undercooked meat, and various other routes of exposure (2, 3). Due to multiple routes of *S. suis* transmission, over 30 distinct serotypes of *S. suis* have been identified based on the antigenicity of their capsular polysaccharide, with serotype 2 being predominantly prevalent in China (4). The predominant approach employed in Chinese pig farms for the management of *S. suis* infections has primarily relied on the use of antibiotic. However, the indiscriminate use of antibiotics has led to the emergence of antibiotic-resistant strains of *S. suis*, including the appearance of multidrug-resistant variants in China (4, 5). Currently, *S. suis* exhibits resistance to a diverse array of antibiotics, including macrolides, lincosamides, aminoglycosides, chloramphenicol, β-lactams, fluoroquinolones, and tetracyclines (5), which poses substantial challenges for therapeutic strategies and needs reevaluation of the antibacterial drugs (6). The increasing resistance underscores the need for improved surveillance and careful antibiotic use in swine farming (7). Concurrently, an urgent demand exists for formulating preventive and control strategies in China, as well as for developing novel antimicrobial agents with multitarget capabilities. These advancements are crucial for strengthening our defenses against the burgeoning threat posed by antimicrobial-resistant strains in China.

In this minireview, we conduct a comprehensive analysis of the epidemiological patterns and therapeutic approaches for *S. suis* infections in China, encapsulating recent trends in the epidemiology of *S. suis* in swine populations, agricultural settings, and human cases within the country. This provides an overarching synthesis of the transmission dynamics associated with resistant strains. The aim of this work is to elucidate the distribution patterns of antibiotic-resistant *S. suis*, thereby informing effective control measures and facilitating the development of novel antibacterial therapies for this significant zoonotic pathogen.

## PREVALENCE OF PORCINE *STREPTOCOCCUS SUIS* WIDESPREAD

To analyze the influence of *S. suis* prevalence in swine, an in-depth research of the global spread of *S. suis* is conducted. Initially, the complex transmission pathways in China are explored, including environmental factors, animal contact, and the importance of virulence factors in disease progression. This segment further explores the pressing concern of antibiotic resistance in *S. suis* strains. Additionally, the analysis of the genetic evolution and resistance gene dynamics of Chinese *S. suis* strains is conducted to elucidate their wider impact on public health and agriculture.

### Identification analysis of *Streptococcus suis* worldwide

The rates of *S. suis* can be attributed to various factors, such as livestock density and agricultural methods, leading to higher frequencies in swine herds (8). Recent trends have revealed a relationship between the isolation of *S. suis* and agricultural practices (9, 10). In that case, the farming practices in different regions can affect the isolation rate of *S. suis*. In Asian countries such as South Korea and Thailand, the detection rate of *S. suis* on pig farms exceeds 50% (11, 12). Similarly, in China, the prevalence of *S. suis* on pig farms is generally greater than 40% (13), owing to the extensive practice of small-scale pig farming across Asia. This practice heightens the risk of human-pig interactions and the transmission of *S. suis*, along with the potential development of antimicrobial resistance (14).

**Fig 1:**
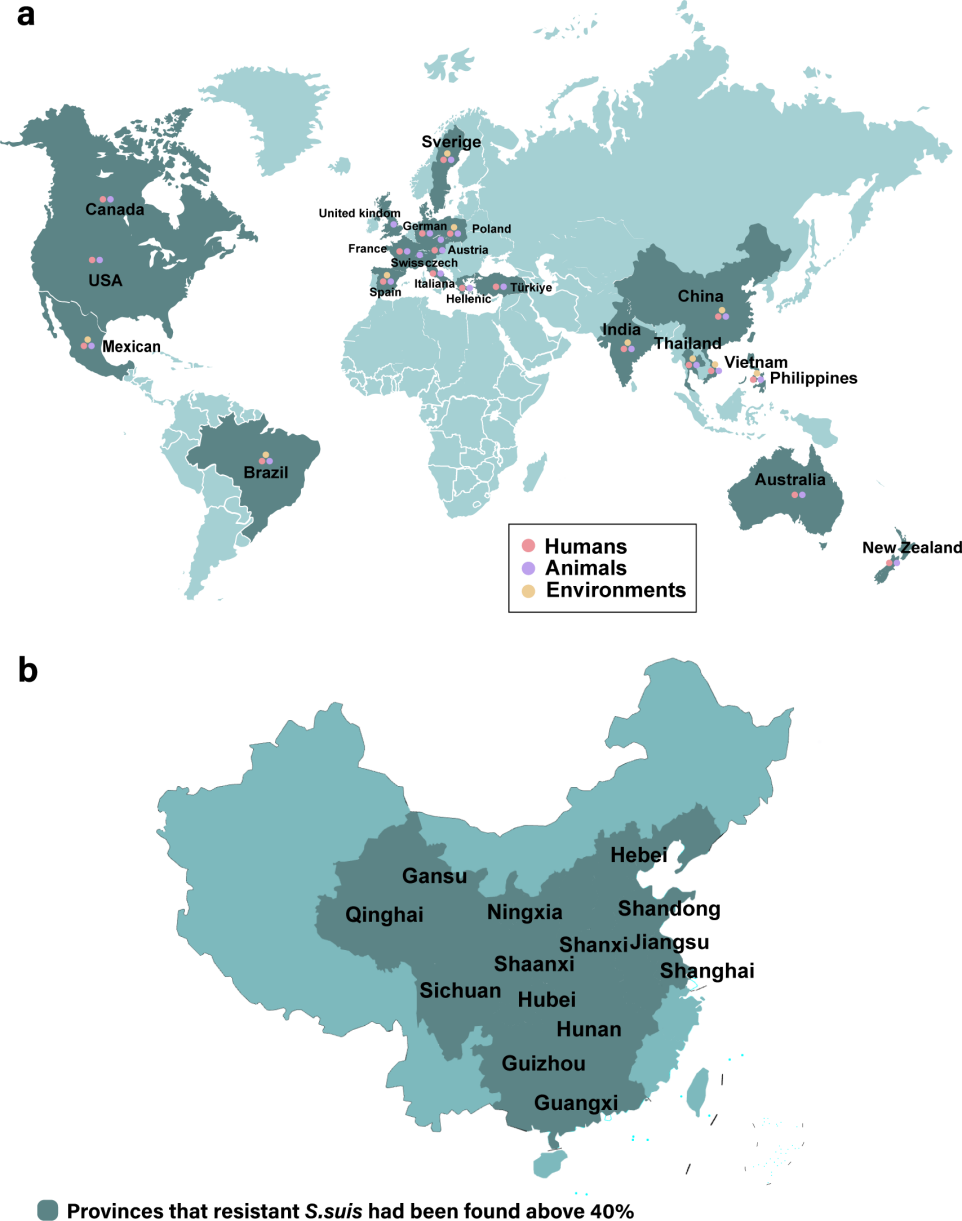
Geographical and temporal distribution of resistant *Streptococcus suis*. (**a**) Global isolation of *erm*/*tet* drug resistance genes from *S. suis* in humans, animals, and environmental samples (11, 12, 23–25). (**b**) Chinese provinces with documented cases of antimicrobial-resistant porcine *S. suis* (13, 26–36). Source: Standard Map Service website of the Ministry of Natural Resources of China.

In contrast, infections are more frequently associated with direct contact with live pigs in Europe (15). Switzerland presents an intriguing comparison because of its lower rate of pig export and transportation, coupled with a unique pig farming structure (16). These factors potentially elucidate the notably lower incidence of human *S. suis* infections compared with those of countries like Spain and the Czech Republic (17, 18). Furthermore, there are clear regional differences in *S. suis* infection; STs 1/2 and 2 are more prevalent in the Americas (19, 20), whereas STs 1 and 7 are commonly found in European countries. Australia tends to observe similar serotypes as the UK and North America (21, 22). The infection rates in these outbreaks exceed 90%, indicating a widespread and significant health concern within the swine industry (Fig. 1a).

There is an increasing trend in antibiotic resistance within these isolates, posing the challenges to the management of *S. suis* infections. From 2011 to 2018, *S. suis* pathogens from Australian pig farms were resistant to tetracycline and erythromycin (37). Similarly, pathogen isolates from Swedish pig farms in 2020 demonstrated resistance to tetracycline, with some strains demonstrating resistance to enrofloxacin (11, 38). The emergence of resistant *S. suis* strains has been attributed to the inappropriate and prolonged use of antibiotics (39, 40). Hence, the key challenge lies in achieving a harmonious equilibrium between pork production and the prudent utilization of antibiotics, particularly in China.

The identification of *S. suis* strains underscores the urgent need for rigorous monitoring and proactive management strategies to mitigate potential outbreaks and protect public health (41), particularly in China, which possesses the largest swine herds globally. A recent study revealed that nearly all pathogenic *S. suis* isolates from major pig farms in China exhibited resistance against eight broad-spectrum antibiotics (42). Infections attributed to *S. suis* had been documented across various regions of China, with notably higher incidence rates in the eastern provinces. Most strains can be categorized as serotype 2 (13). This consistency in strain type emphasizes the role of animal product movement in the dissemination of infectious agents. In Jiangxi Province, the isolation rate of *S. suis* from 314 nasal swab samples was 34.08%. These findings indicate that nearly 80% of the *S. suis* strains was resistant to several antibiotics, including vancomycin, penicillin, minocycline, and chloramphenicol. These findings indicate the significant presence of antimicrobial-resistant strains of *S. suis* in the region (43). Pig farms in Heilongjiang showed more than 80% of *S. suis* isolates resistant to antibiotics such as erythromycin, tetracycline, and chloramphenicol (42) (The provinces in China which had more than 40% of resistant *S. suis* has been mentioned in Fig. 1b) (13, 26–36). Furthermore, interregional transportation of pig products plays a crucial role in the dissemination of homogenous strains, especially serotype 2 strains, which are prevalent in outbreaks due to their virulence and resistance characteristics (44).

The extensive evidence underscores the widespread prevalence of *S. suis* and the emergence of drug-resistant strains in China, emphasizing the urgent need for comprehensive monitoring and strategic interventions.

### Epidemic characteristics of *Streptococcus suis* in China

*Streptococcus suis* is highly prevalent in densely populated livestock areas with intensive farming (45, 46), particularly in China, a leading global pork producer. The elevated prevalence and extended incubation period of *S. suis* in porcine populations pose challenges to the livestock sector (47, 48) and present serious risks to public health (49). The outbreaks of *S. suis* in China between 1998 and 2005 raised concerns about the potential for widespread infections. Continued reports of *S. suis* cases in Guangxi from 2007 to 2018 further emphasize the persistent risk of this zoonotic infection (35). Furthermore, *S. suis* has exceptional survival capabilities across diverse environmental conditions, aiding its transmission between animals and humans (50). The diversity of virulence factors and serotypes in *S. suis* complicates vaccine development (51, 52). Luckily, the extensive genomic analyses have been conducted on human-derived ST1 strains of *S. suis* in China, providing insights into its virulence through typing and assessment (13, 53). This evidence highlights the importance of studying the epidemic characteristics of porcine *S. suis* to strengthen infection prevention in China.

### Infectious route of *Streptococcus suis* in China

**Fig 2:**
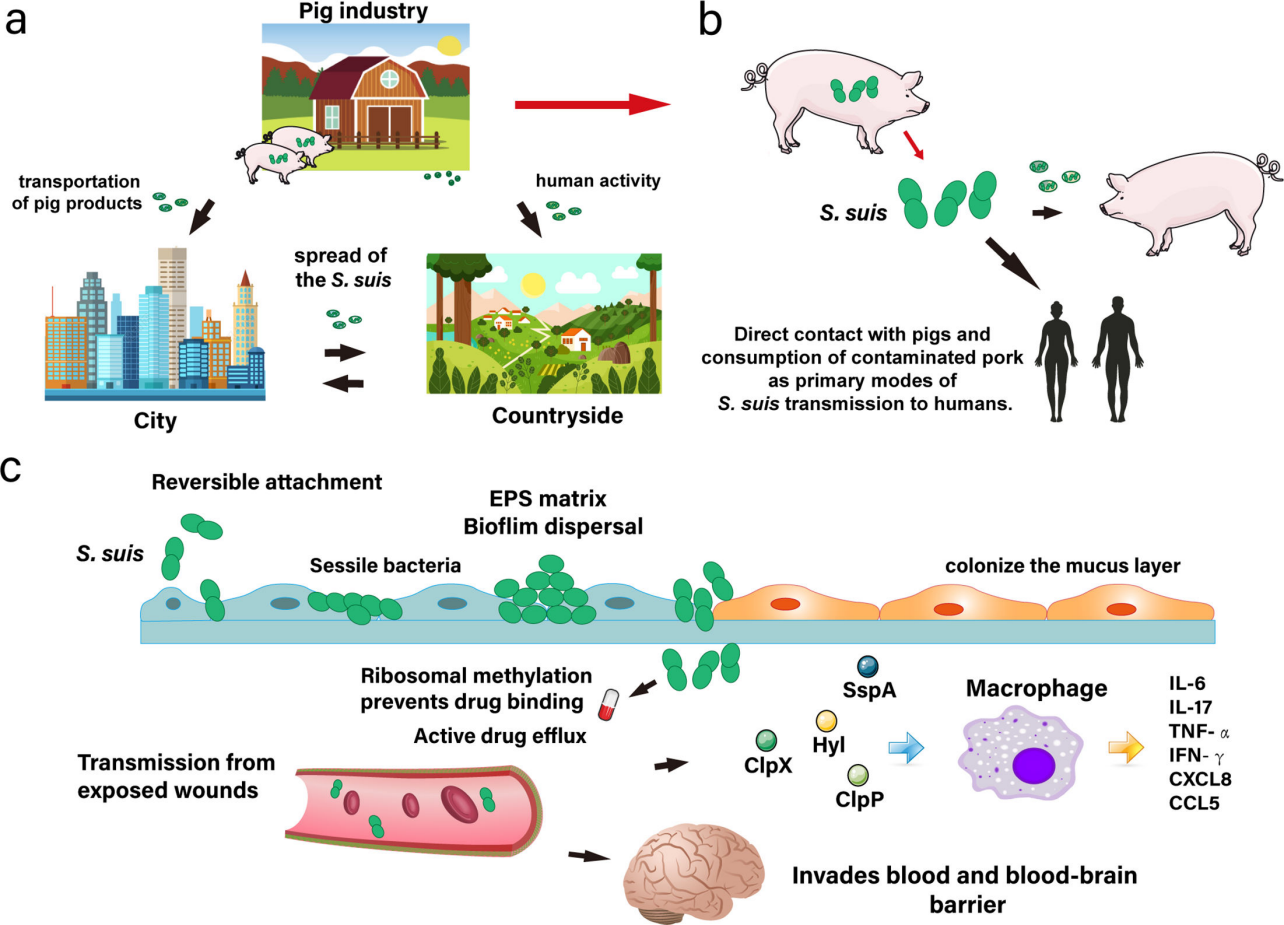
Transmission route of porcine *Streptococcus suis*. (**a**) Porcine *S. suis* can be transmitted among pigs orally and to humans through contact with wounds or the consumption of undercooked pork. (**b**) Bacteria can persist in environmental reservoirs such as soil and water, posing an infection risk to individuals who handle pigs or raw pork in both rural and urban settings. (**c**) Virulence factors enable *S. suis* to adhere to host cells, circumvent immunological defenses, and penetrate the bloodstream and the blood-brain barrier, leading to the activation of macrophages and the release of proinflammatory cytokines.

*S. suis* is recognized as an occupational hazard, primarily impacting swine industry workers in many Organization for Economic Cooperation and Development (OECD) nations, where it is regarded as a significant pathogen responsible for human disease (54). The inadequate quality of food safety and contact with raw pork products in markets have been regarded as potential sources of human infection (55) (Fig. 2b).

During *in vivo* infection, *S. suis* effectively colonizes mucosal layers and is enhanced by the formation of bacterial biofilms (56). As bacteria breach the mucosal layer, they utilize adhesins and invade surfaces (57). *S. suis* can access the bloodstream throughout the host organism (58) and cross the blood-brain barrier (BBB), resulting in meningitis (59, 60). Research indicates that O-acetyl-serine sulfhydrylase (OAHS) enhances the pathogenicity of *S. suis* by increasing the permeability of the host BBB and facilitating immune evasion (59). Similarly, enolase (Eno) from SS2 has been identified as a virulence factor capable of compromising the integrity of the BBB. This functionality stresses the critical role of Eno in infection pathogenesis, with barrier penetration being a critical step (61) (Fig. 2c).

Adhesins of *S. suis* are critical for tissue invasion (62). Critical adhesins from SS2, including elongation factor thermo unstable, enolase, lactate dehydrogenase, and fructose-bisphosphate aldolase, have been shown to interact with host fibronectin. This interaction is crucial for establishing bacterial adhesion, a precursor to the invasion of host tissues (63). Meanwhile, the autolysin AtlA in *S. suis* (AtlASS) has been identified as a novel cell surface protein, characterized by a unique glycine-tryptophan (GW) module and an N-acetylmuramidase domain, suggesting its specialized role in bacterial cell wall remodeling. This feature suggests its potential role in bacterial physiology and pathogenicity (64). Furthermore, research has revealed that the fatty acids facilitate the bacterial invasion of the host. Additionally, lipoteichoic acids have been identified in three SS2 strains, which exhibit distinct genetic backgrounds and varying levels of virulence (65). *S. suis* also produces toxins, including suilysin (SLY) and exotoxins, which disrupt the host cell membrane and facilitate bacterial invasion and dissemination (Fig. 2c). Additional studies have confirmed that SLY-induced inflammasome activation plays a crucial role in the development of Space-Time Scan Statistics (STSLS), mediating inflammatory responses and disease outcomes (66). High expression of SLY in *S. suis*, particularly in nonepidemic strains, is sufficient to induce NLRP3 inflammasome hyperactivation. This leads to cytokine storms and contributes to the severity of STSLS (67). Researchers also suggest that NLRP3 contributes to cytokine production, resulting in toxic shock-like symptoms during infection with epidemic strains of *S. suis* (68) (Fig. 4b).

### Transformation of resistance genes in China

The dynamics of *S. suis* in Chinese swine and human populations emphasize not only the intricacies involved in controlling this pathogen but also the crucial role of genetic transformation in its proliferation and persistence. *S. suis* strains with specific clonal complexes demonstrate strong pathogenicity (69). The *S. suis* isolates from China, which had specific sequence types, significantly influence their pathogenicity (70). Understanding the gene composition of *S. suis* is essential for elucidating the mechanisms by which this pathogen spreads resistance genes among hosts.

**Fig 3:**
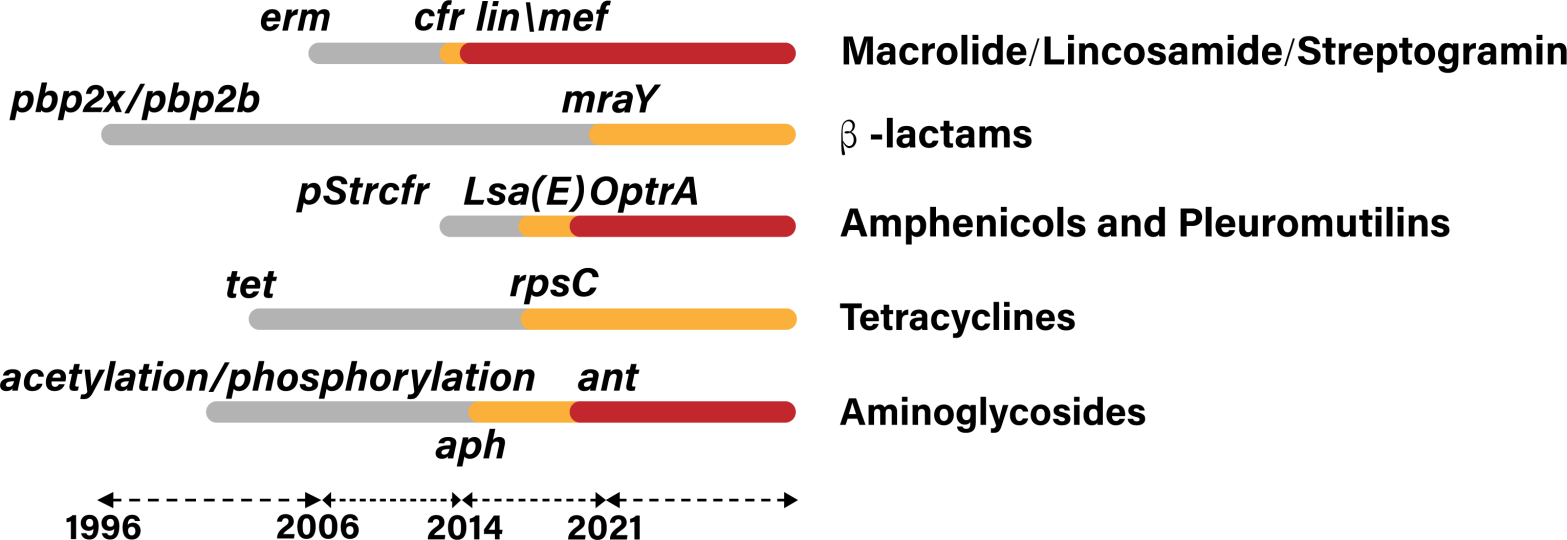
Timeline illustrating the emergence and development of antimicrobial-resistant *S. suis* gene variants worldwide since 1996 (14, 72, 73).

Recent studies have investigated the antibiotic resistance profiles and molecular characteristics of *S. suis* isolates from both swine and human sources in China, providing valuable insights into zoonotic transmission and resistance mechanisms (71). This research offers insights into the resistance mechanisms and genetic diversity of the isolates, highlighting the implications for public health and the zoonotic potential of these bacteria (71). Genomic sequencing of *S. suis* explores the evolutionary dynamics of resistance traits and highlights significant implications for global public health and biosecurity strategies (14). For example, the presence of *erm*(B) genes and *tet*(O) genes was identified in 436 *S*. *suis* isolates collected from 20 provinces across China between 2011 and 2019. Another report described the presence of the *optr*A gene in strains isolated from nasal samples on pig farms in six provinces of China between 2016 and 2018. In addition, it also examines the genetic evolution of *S. suis* in healthy pigs by analyzing capsular serotypes and the presence of oxazolidinone resistance genes, suggesting potential transmission paths and evolutionary pressures on antimicrobial resistance development (72). The identification and characterization of *Streptococcus* strains from a pig farm in Guangdong revealed antimicrobial resistance profiles, including the resistance gene *tet*(M), with selective pressures from agricultural antibiotic use, as well as the presence of the *tet*(O) gene (73) (Fig. 3).

A comprehensive analysis of 366 clinical *S. suis* isolates from China was conducted to construct an evolutionary dendrogram. The results revealed that *S. suis* type ST7, which is commonly found, possesses several antibiotic resistance genes, such as *tet* (O), *mef* (A), *erm* (B), *msr* (D), and *aph* (3′)-III (74). Environmental and antibiotic pressures drive the genetic evolution of a new *S. suis* clade, promoting its adaptation and impacting human health. Chinese studies have also examined if the *S. suis* evolutionary adaptations are driven primarily by ecological pressures and their inherent mechanisms to prosper in diverse environments, thereby influencing its epidemiological trends within the area (75). Fortunately, Chinese researchers had explored the genetic differentiation of serotype 8, uncovering novel genes implicated in its virulence and significantly advancing our comprehension of its pathogenic mechanisms (76). By all these aspects, our analysis provides a comprehensive understanding of pathogenic resilience and the substantial challenges it presents to public health and agricultural stability in China.

## PREVENTION AND CONTROL OF RESISTANT *STREPTOCOCCUS SUIS*

In light of extensive research on the epidemiology and antimicrobial resistance of *S. suis* in China, the implementation of targeted prevention and control strategies is crucial. These measures should include promoting prudent antibiotic use, developing effective vaccines and exploring novel drug targets and treatments to mitigate resistant strains. Together, these initiatives are crucial for addressing the growing threat of this pathogen in China.

### Rational use of antibiotics and vaccines in China

China has implemented rigorous measures to promote the prudent use of antibiotics and vaccines, aiming to combat resistance and enhance public health. These endeavors encompass stringent regulatory frameworks, extensive educational initiatives directed at healthcare professionals and the general populace, and continuous surveillance to assess efficacy and adherence.

Since 2020, China has prohibited the use of antibiotics as growth promoters in animal feed to safeguard the therapeutic efficacy of these drugs and mitigate the risk of bacterial resistance arising from prolonged exposure to antibiotics (77). This policy prohibits specific antibiotics, including bacitracin zinc and virginiamycin premixes. As a result of this policy, there has been a notable reduction in the prevalence of the colistin resistance gene *mcr*-1 among animals and humans (78). Although the use of therapeutic antibiotics may increase in the short term, the reduction in antibiotic selection pressure will lead to a decrease in resistance issues in the long term (79, 80). Concurrently, a pilot program was launched aiming to eradicate antibiotics in livestock feed entirely, prompting feed companies to explore alternative additives such as acidifiers and enzymes, enhancing nutrition without antibiotics and pushing industry innovation (81, 82).

**Fig 4:**
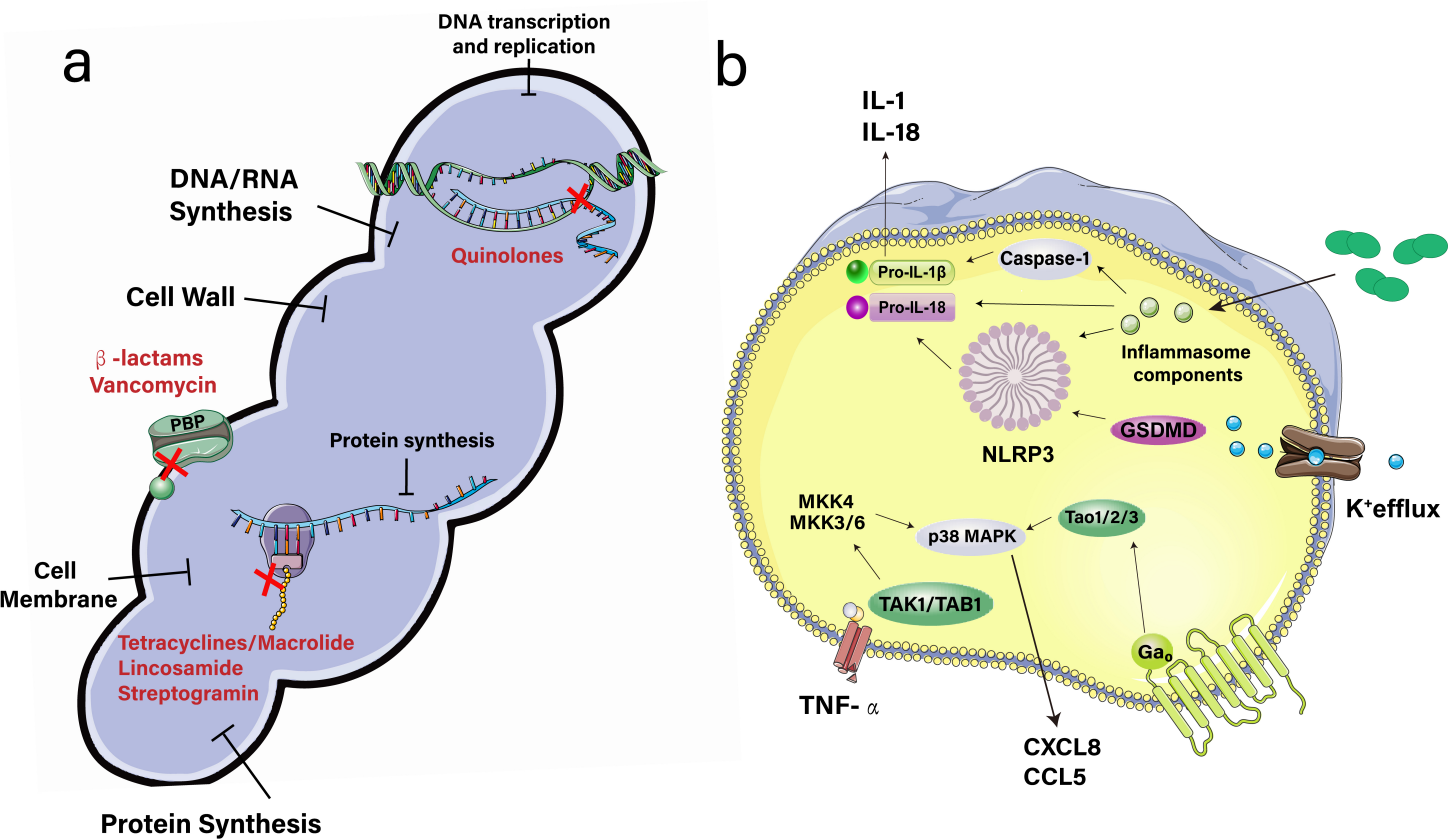
Mechanisms of antimicrobial resistance and pro-inflammatory signaling pathways to fight *Streptococcus suis* resistance. (**a**) Schematic representation of the resistance mechanisms employed by porcine *S. suis* strains, demonstrating how these strains undermine host immune defense mechanisms. (**b**) A diagrammatic overview of the host immune response pathways, identifying molecular targets for the development of innovative therapeutic agents intended to neutralize resistant *S. suis* strains.

Penicillin is the preferred antibiotic for the treatment of *S. suis* infections, particularly those with systemic manifestations (83). A report highlights how antibiotic utilization in China contributes to resistance development, emphasizing the role of mobile genetic elements in the spread of resistance genes among bacterial populations (13). However, an additional study unveiled the repercussions of antibiotic administration in China, illustrating how such practices facilitate the spread of resistance via mobile genetic elements (77). Research highlights the urgent need to develop effective strategies for managing infections that avoid exacerbating antibiotic resistance. The historical overuse of antibiotics in human and veterinary domains in China poses a significant challenge (84) (Fig. 4a).

Several vaccines have been developed to prevent *S. suis* infection in China. For example, novel vaccines that target immune membrane-associated proteins of *S. suis* and a gene deletion vaccine (85) have been developed. In addition, researchers have explored the use of combined immunoinformatic methods and evaluated a multiepitope vaccine candidate against *S. suis* infection (86). They utilize advanced bioinformatics tools to identify and assemble the most promising epitopes into a vaccine formulation.

Alongside vaccine development, the implementation of rapid detection methods can effectively control the spread of *S. suis* infection. A rapid colloidal gold immunochromatographic assay has been developed for detecting *S. suis* using the HtpsC protein to identify the pathogen (87). Researchers have introduced two loop-mediated isothermal amplification assays for the rapid detection of the *erm*B and *mef*A genes in *S. suis* (88). These approaches enhance the ability to promptly identify resistance to macrolides, providing a valuable tool for monitoring and managing antibiotic resistance in clinical settings.

### Novel potential targets

Recent reports have focused on identifying novel biological targets that combat *S. suis* infections, including the treated targets of unique virulence factors and specific regulatory proteins.

A study identified SssP1, a fimbria-like protein of *S. suis*, as a promising candidate for therapeutic intervention because of its interaction with vimentin and potential impact on bacterial meningitis (89). In addition, investigations into the impact of metformin on the LuxS/AI-2 quorum-sensing system and biofilm formation in *S. suis* suggest that metformin may disrupt communication during biofilm development in this pathogen, identifying a potential new target for the development of therapeutic strategies to prevent and manage *S. suis* infections (90). This approach presents a novel perspective for addressing bacterial virulence and resistance. Moreover, a study elucidated the role of RbpA, a protein found in SS2, as a crucial global regulator of its pathogenicity (91). This identification of RbpA highlights its potential as a novel therapeutic target, paving the way for innovative approaches in the development of strategies to manage infections induced by this pathogen. Finally, one study utilized transposon library screening to identify genes associated with biofilm formation in *S. suis*, revealing targets that may enhance treatment strategies (92). This innovative approach highlights the importance of disrupting biofilm processes to combat infections effectively (Fig. 4b).

Currently, the identification of multiple potential therapeutic targets provides insights into the discovery and development of host-acting agents to combat *S. suis* infections. Autolysins and porcine lysins (SLY) have immunogenic effects on *the S. suis* biofilm-associated protein PDH (93). These discoveries open up potential pathways for developing treatments specifically targeting this interaction to prevent infections. Certain proteins from *S. suis*, such as endopeptidase O and OAHS, bind plasminogen via its protein endopeptidase O, aiding in the evasion of the immune system (82).

Inhibiting the adhesion or internalization of pathogens is a critical and effective strategy for combating *S. suis* infections. Targeting these initial stages of infection allows for the design of specialized inhibitors and vaccines that not only suppress resistance but also improve therapeutic outcomes.

### Alternative approach to antibiotics

In addition to the rapid detection in combating *S. suis* infections, the judicious utilization of antibiotics for treatment holds equal significance. The strategy of employing combination therapy is widely adopted to suppress infections caused by *S. suis*, which is assisting in mitigating the development of antibiotic resistance.

The study of AVPL, a phage lysin from *Aerococcus viridans*, illustrates its potential for targeting and lysing *S. suis*, providing a sustainable alternative to traditional antibiotics (94). Methyl anthranilate can disrupt *S. suis* biofilms and weaken polysaccharide defenses, potentially reducing the need for higher antibiotic doses and addressing resistance issues (95). In addition, reports have evaluated the role of the glycerol repressor GlpR in enhancing *S. suis* resistance and virulence, indicating that targeted therapies can reduce the dependence on broad-spectrum antibiotics and address resistance (96). This study revealed that exogenous methionine can reverse *S. suis* resistance to macrolides, providing a strategy to increase antibiotic effectiveness and mitigate resistance (97).

In addition to the prudent utilization of existing antibiotics, the management of *S. suis* infections can also be achieved through the discovery of novel compounds. For example, 25-hydroxycholesterol is a compound that targets membrane remodeling and reduces bacterial internalization by altering membrane cholesterol (98). The synergistic combination of conventional antimicrobials and essential oils has been shown to be more effective at inhibiting *S. suis* than the use of only AMBs (99). Empirical evidence from *in vitro* killing curves and *in vivo* treatment trials suggests that combination regimens, such as ampicillin with ampicillin and timolol with macrolide, demonstrate superior therapeutic effects on *S. suis* compared with single antibiotic use (100).

The foundational principles of traditional Chinese medicine focus on fortifying the body and eradicating illness, adopting a holistic approach that encompasses the host, the pathogen, and treatment. This perspective provides intriguing potential for the development of new pharmaceuticals aimed at combating antibiotic-resistant bacteria by bolstering host immunity (101). Recent studies have utilized host-acting antibacterial compounds (HACs) as multitarget therapeutic strategies to combat resistant bacteria (6). Among HACs, herbal compounds exhibit host-acting therapeutic effects against resistant bacterial infections. The use of paeoniflorin in conjunction with the antibiotic norfloxacin can not only increase the antibacterial efficacy of norfloxacin but also reduce *S. suis* resistance to drugs (102). Epigallocatechin-3-gallate (EGCG), a major polyphenol found in green tea, has inhibitory effects on sortase A, which is an enzyme crucial for the virulence of *S. suis*. EGCG can disrupt the function of sortase A, thereby potentially reducing the pathogenicity of *S. suis* (103). Certain ingredients in traditional Chinese medicines, such as flavonoids, have displayed significant potential antibacterial activities, and our laboratory research has previously demonstrated the strong anti-*S*. *suis* hemolysin effects of Chinese medicines rich in flavonoids (104, 105). The primary mechanism of action of these compounds is the inhibition of the coding and transcription of the hemolysin gene (106, 107). These findings have important implications for the potential integration of Chinese medicinal components in future treatment strategies for *S. suis*. Green tea polyphenols can influence significant metabolic pathways in *S. suis*, including pyrimidine metabolism, protein digestion, and absorption (7). Flavonoids can decrease the activity of the virulence factor (SLY), thereby reducing the production of inflammatory factors and cell autophagy in *S. suis* (86). These novel phototherapeutic approaches provide valuable insights into the prevention and management of drug-resistant *S. suis* infections.

## CONCLUSION AND PERSPECTIVE

This minireview addresses the significant challenges presented by the zoonotic pathogen porcine *S. suis*, which is a threat to both public and animal health on a global scale. The epidemiology of *S. suis*, influenced by its transmission, dietary sources, environmental, and host interactions, requires further study and poses the formulation of effective treatments. Recent researches have highlighted the prevalence of *S. suis* infections, particularly within large-scale pig farming operations worldwide, where infection rates frequently exceeded 40%, with a notable predominance of serotype SS2 observed in China (4).

Historically, antibiotic therapies have played a pivotal role in the management of *S. suis* infections. However, increasing concerns regarding drug resistance and antibiotic residues necessitate the exploration of novel therapeutic approaches. It is imperative that ongoing research and development efforts prioritize the discovery of multitarget antibacterial drugs, which may offer a more sustainable and effective response to *S. suis* infections. Several promising drug targets have been identified, indicating that alternative strategies could substantially mitigate both the prevalence and impact of these infections.

Future initiatives aimed at combating *S. suis* should capitalize on an enhanced understanding of its transmission dynamics and resistance mechanisms. The integration of advanced genomic techniques alongside machine learning holds great potential for obtaining deeper insights into pathogen behavior and resistance patterns, thereby facilitating innovative intervention strategies. Prioritizing multitarget drugs and alternative therapeutic modalities will be essential for effectively managing *S. suis* infections, mitigating the spread of resistance, and safeguarding public health as well as safety of both human and animal populations. Continued vigilance and proactive research are imperative to adapt to the evolving dynamics of this pathogen and to protect global health.
